# Quality of Cohort Studies Reporting Post the Strengthening the Reporting of Observational Studies in Epidemiology (STROBE) Statement

**DOI:** 10.4178/epih/e2011005

**Published:** 2011-06-07

**Authors:** Jalal Poorolajal, Zahra Cheraghi, Amin Doosti Irani, Shahab Rezaeian

**Affiliations:** 1 Research Center for Health Sciences and Department of Epidemiology & Biostatistics, School of Public Health, Hamadan University of Medical Sciences, Hamadan, Iran.; 2 Department of Epidemiology & Biostatistics, School of Public Health, Hamadan University of Medical Sciences, Hamadan, Iran.

**Keywords:** STROBE statement, Cohort studies, Observational studies, Reporting

## Abstract

The quality of reporting of cohort studies published in the most prestigious scientific medical journals was investigated to indicate to what extent the items in the Strengthening the Reporting of Observational Studies in Epidemiology (STROBE) checklist are addressed. Six top scientific medical journals with high impact factor were selected including New England Journal of Medicine, Journal of the American Medical Association, Lancet, British Medical Journal, Archive of Internal Medicine, and Canadian Medical Association Journal. Ten cohort studies published in 2010 were selected randomly from each journal. The percentage of items in the STROBE checklist that were addressed in each study was investigated. The total percentage of items addressed by these studies was 69.3 (95% confidence interval: 59.6 to 79.0). We concluded that reporting of *cohort* studies published in the most prestigious scientific medical journals is not clear enough yet. The reporting of other types of observational studies such as case-control and cross-sectional studies particularly those being published in less prestigious journals expected to be much more imprecise.

## INTRODUCTION

Randomized trials are generally considered the gold standard for evaluating both the efficacy and the potential side effects of new therapeutic or preventive interventions in both clinical medicine and public health [1]. However, much of the biomedical research into the cause of diseases comes from observational studies [2]. The results of these studies should be reported as transparently as possible "*so that readers can follow what was planned, what was done, what was found, and what conclusions were drawn*" [3]. Unfortunately, reporting of observational research is neither precise nor clear enough to assess the strengths and weaknesses of the evidence [4, 5].

To improve the reporting of observational research, a group of experts developed a checklist of items known as the Strengthening the Reporting of Observational Studies in Epidemiology (STROBE) statement [6]. The STROBE statement was developed in 2007 to assist authors in report writing of observational studies, including cohort, case-control, and cross-sectional studies, to support editors and reviewers in considering such manuscript for publication, and to help readers in appraising published articles critically [3].

Concerning valuable recommendations made in this statement, expected that reporting of observational studies published after 2007 being improved enough to assess the strengths and weaknesses of the evidence. The present study was set up to investigate the quality of reporting of cohort studies published in the most prestigious scientific medical journals and to indicate to what extent the items in STROBE checklist are noticed by both authors and publishers.

## METHODS

We conducted a cross-sectional study and selected six top scientific medical journals with high impact factor (IF) among the most prestigious and important medical journals indexed in international databases. These included New England Journal of Medicine (N Engl J Med) (IF 50.017), Journal of the American Medical Association (JAMA) (IF 31.718), Lancet (IF 28.409), British Medical Journal (BMJ) (IF 12.827), Archives of Internal Medicine (Arch Intern Med) (IF 9.110), and Canadian Medical Association Journal (Can Med Assoc J) (IF 7.464). We were to include Nature and Annals of Internal Medicine in this survey, but they were not open access journals.

Among the observational studies, cohort studies are much more expensive and take longer follow-up time than case-control and cross-sectional studies. Therefore, the results obtained from cohort studies are of substantially superior quality to other observational studies. In fact, cohort studies are considered the gold standard in observational epidemiology. Accordingly, we selected cohort studies, either prospective or retrospective, for this evaluation.

We randomly selected 10 cohort studies published in each of the six medical journals from January to August 2010. To do so, we sorted the articles from newly published articles to the old ones. Then, looked for the cohort studies to find ten eligible articles. If there were not enough cohort studies, we searched second half of 2009 to obtain additional studies. Accordingly, we enrolled 60 cohort studies from six prestigious scientific medical journals. The studies were randomly assigned to four reviewers through drawing lots. The reviewers independently made the decisions on the number of items, from the STROBE checklist, which were addressed in the selected studies. The reviewers were not blinded to the names of the studies' authors and journals.

In order to check the reliability of the four reviewers' judgment on the quality of reporting of cohort studies, we conducted two consecutive pilot studies as follows. First, an article was randomly selected from JAMA. All four reviewers assessed the strengths and weaknesses of the evidence reported in the same article using the predetermined checklist of items. The disagreements were discussed to reach the same perception of the checklist in order to increase the between reviewers reliability. Then, the four reviewers evaluated the quality of reporting of another article that was randomly selected. There was no significant differences between the reviewers' judgment in the second pilot study (p=0.823).

In order to assess the validity of the reviewers' judgments, two reviewers made decision on the quality of reporting of each cohort study independently. Any disagreements were resolved by adjudication with a third author.

The STROBE statement included a checklist of 22 items. In order to estimate the quality of reporting of cohort studies more accurately, we divided the items into 47 sub-items (Table 1). We considered three choices for each sub-item (reported/not reported/not applicable). The percentage of each sub-item addressed in the selected articles was estimated. The total percentage for all sub-items was reported.

All statistical analysis was performed at 95% significant level using statistical software Stata version 10.0 (StataCorp, College Station, TX, USA).

## RESULTS

In this survey, 60 cohort studies were selected for evaluation including 10 studies from the six prestigious scientific medical journals including: N Engl J Med, JAMA, Lancet, BMJ, Arch Intern Med, and Can Med Assoc J. From these, 56 studies were published in 2010 and four studies in 2009. The percentage of items and sub-items addressed by these studies are summarized in Table 1. The sub-items were not applicable in 7.1% (95% confidence interval [CI], 1.3% to 13.0%), were not reported in 23.6% (95% CI, 15.3% to 31.5%), and were reported in 69.3% (95% CI, 59.6% to 79.0%) of the cohort studies.

Of 47 sub-items investigated in this survey, nine sub-items were reported 100%, 22 sub-items were addressed in more than 90% of the studies, 28 sub-items were included in more than 75% of the studies, and 32 sub-items were addressed in more than 50% of the studies.

## DISCUSSION

STROBE checklist of items provides valuable recommendations for both authors to report the results of observational studies clearly as well as for editors, reviewers, and readers to appraise such reports critically [3]. On overall, almost 69.3% of the items and sub-items in STROBE checklist were addressed by cohort studies published in six top scientific medical journals three year after dissemination of STROBE statement.

However, what has happened to the reports of other types of observational studies? The results of the present study represent the reporting of cohort studies published in six prestigious scientific medical journals that generally accept the well-done and well-written studies. However, there are numerous observational studies, the results of which are published in other less fastidious peer-reviewed medical journals. Thus, it is expected that the quality of reporting of such studies is much poorer than what reported in the present study, although the result of present study is not desirable enough. Furthermore, cohort studies are much more expensive and take longer follow-up time than other types of observational studies such as case-control and cross-sectional design. Hence, the reporting of cohort studies is generally expected to be of substantially superior quality to other observational studies. Accordingly, if this survey had been planned to assess reporting of case-control or cross-sectional studies, the estimated result would be much more undesirable.

We found no similar studies but one. Poorolajal et al. [7] conducted a similar study in 2007 to assess the reporting of cohort studies before STROBE statement being issued. However, the design, the results, and the number of sub-items, which were evaluated in that study, was not comparable with that of the present study.

The present study had a number of limitations. First, the limited number of studies evaluated in the present study may increase the possibility of random error. Second, randomly selection of cohort studies from a few prestigious medical journals may increase the possibility of selection bias. Third, the value of all sub-items was not really the same. Hence, adding up all percentages to estimate a summary measure might not be reasonable, although was done to help the readers make an overall judgment.

This result of the present survey represents the quality of the reporting of cohort studies in top scientific medical journals. Hence, we can generalize the results of this survey neither to other types of observational studies nor to the publication of less fastidious peer-reviewed medical journals.

We concluded that reporting of cohort studies published in the most prestigious scientific medical journals is not clear and desirable enough yet. The reporting of other types of observational studies such as case-control and cross-sectional studies particularly those being published in less fastidious peer-reviewed journals is expected to be much more imprecise. This issue should be the focus of the both authors' and editors' special attention when reporting and/or reviewing the reports of observational studies.

## Figures and Tables

**Table 1:**
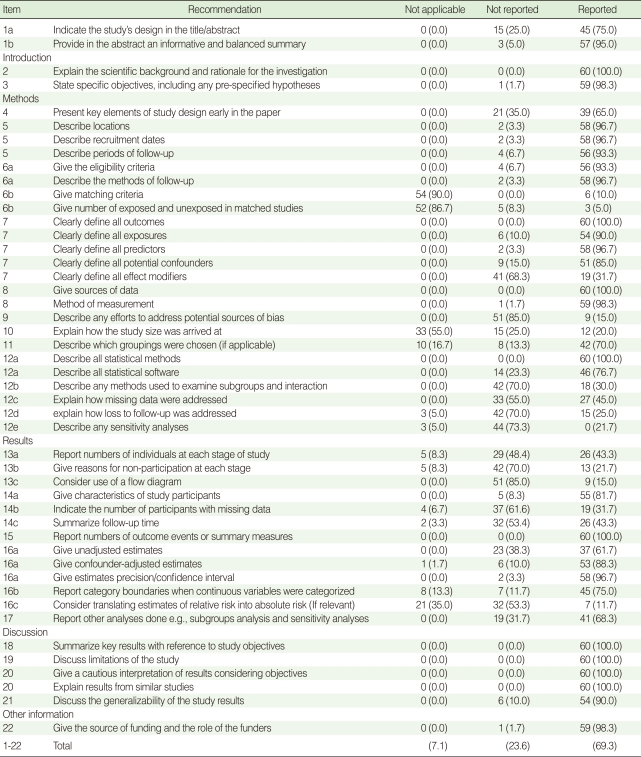
Percentage of items in STROBE checklist which were addressed in reports of cohort studies published in six top scientific medical journals in 2010 (n (%))

STOBE, The Strengthening the Reporting of Observational Studies in Epidemiology.
